# Histological transformation to gliosarcoma with combined BRAF/MEK inhibition in *BRAF V600E* mutated glioblastoma

**DOI:** 10.1038/s41698-023-00398-5

**Published:** 2023-05-25

**Authors:** Blessie Elizabeth Nelson, Neha K. Reddy, Jason T. Huse, Behrang Amini, Mirella Nardo, Mohamed Gouda, Shiao-Pei Weathers, Vivek Subbiah

**Affiliations:** 1 Department of Investigational Cancer Therapeutics, The University of Texas MD Anderson Cancer Center, Houston, TX USA; 2 Department of Internal Medicine, The University of Texas at Austin, Austin, TX USA; 3 Department of Pathology and Translational Molecular Pathology, The University of Texas MD Anderson Cancer Center, Houston, TX USA; 4 Department of Diagnostic Radiology, The University of Texas MD Anderson Cancer Center, Houston, TX USA; 5 Department of Neuro-Oncology, The University of Texas MD Anderson Cancer Center, Houston, TX USA; 6 Division of Pediatrics, The University of Texas MD Anderson Cancer Center, Houston, TX USA; 7 MD Anderson Cancer Network, The University of Texas MD Anderson Cancer Center, Houston, TX USA

**Keywords:** CNS cancer, Molecular medicine, Oncogenesis

## Abstract

The identification of *BRAF V600* mutation in multiple cancers beyond melanoma and the development of combined *BRAF* and *MEK* targeting agents have altered the landscape of tissue-agnostic precision oncology therapies with an impact on survival outcomes. Despite initial efficacy, resistance emerges, and it is pertinent to identify putative resistance mechanisms. We report a case of recurrent glioblastoma (GBM) harboring *BRAF V600E* alteration who initially responded to combined BRAF + MEK inhibition and subsequently developed treatment resistance by histological transformation to gliosarcoma and acquisition of oncogenic *KRAS* ^*G12D*^ *and an NF1* ^*L1083R*^ mutation. This documented case represents an initial evidence of a developing phenomenon in cancer research as it provides the first evidence of an emergent KRAS G12D/NF1 L1083R aberration with histological transformation occurring concurrently with primary BRAF V600E-altered glioblastoma as a previously unrecognized acquired mechanism of resistance in the setting of combined BRAF and MEK inhibition. This novel finding not only sheds new light on the RAS/MAPK pathway but also highlights the potential for morphological transformation to gliosarcoma, underscoring the critical need for further investigation in this area.

## Introduction

*v-raf murine sarcoma viral oncogene homolog B1 (BRAF)* is a *Rapidly accelerated fibrosarcoma (RAF)* kinase that is activated by *Rat sarcoma virus* (*RAS)* and activates the *mitogen-activated protein kinase kinase (MEK)* pathway. It has emerged as a major oncogenic driver and agnostic target in a wide variety of solid tumors and hematological malignancies^1^. *BRAF* signaling is instrumental for cell growth and activating *BRAF* mutations stimulate pro-oncogenic processes. Over 90% of activating *BRAF* mutations in cancer cells occur within the kinase domain at amino acid *V600*, most commonly resulting in *V600E* mutation^2,3^. Combined *BRAF* and *MEK* inhibition is now the standard of care in melanoma, non-small cell lung cancer, and anaplastic thyroid cancer^4–6^. Recently the combination of Dabrafenib + Trametinib received the Food and Drug Administration (FDA) approval in adult and pediatric solid tumors (except colorectal cancer) based on a demonstration of anti-tumor activity in more than 20 different cancer histologies including *BRAF V600* mutant low-grade glioma (LGG) and high-grade glioma (HGG)^6–8^. However, a greater impetus to identify mechanisms of *BRAF* inhibitor resistance is emerging to guide treatment strategies. Resistance mechanisms to *BRAF* and *MEK* inhibition beyond melanoma have been reported in lung and thyroid cancer however, this has been infrequently described in gliomas^9,10^. Lehmann et al reported on pediatric HGG where increased activation of the *RAS/ Extracellular signal-regulated kinase (ERK)* caused alterations in *RAS* signaling through *Neurofibromatosis type 1 (NF1)* missense mutations. *Phosphatidylinositol-4,5-bisphosphate 3-kinase catalytic subunit alpha (PI3K)/Protein kinase B (AKT)* signaling is also indicted in *BRAF* targeted resistance secondary to aberrations in *PIK3C2G*^11,12^.

In this paper, we report a case of recurrent glioblastoma (GBM) harboring *BRAF V600E* alteration who initially responded to *BRAF* and *MEK* inhibition and subsequently developed treatment resistance by histological transformation to gliosarcoma and acquired an emerging *KRAS* ^*G12D*^ and *NF1* ^*L1083R*^ mutations. To our knowledge, this is the first documented case of emergent *KRAS* ^*G12D*^ and *NF1* ^*L1083R*^ aberration concurrently occurring with primary *BRAF* V600E altered glioblastoma as an acquired mechanism of resistance in the setting of *BRAF* and *MEK* inhibition leading to morphological transformation to gliosarcoma.

## Results

### Case report

**Fig. 1:**
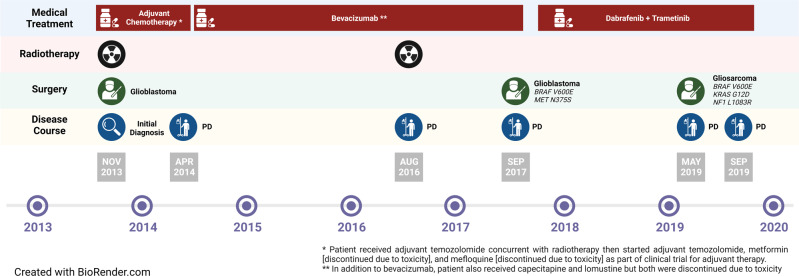
Treatment timeline. Timeline summarizing the treatment course of the patient, including all systemic, surgical, and radiation therapies received.

A female patient in her 30 s presented with a five-day history of headaches, slurred speech, and dyscoordination in fine motor skills. Baseline imaging of the brain via MRI with contrast demonstrated a 3.3 cm ring-enhancing lesion in the right frontoparietal subcortical white matter, suspicious for a glioma. A timeline of the patient’s clinical course and management is depicted in Fig. 1.

She underwent craniotomy with gross total resection and histology was confirmed as glioblastoma. Immunohistochemistry testing was negative for Isocitrate Dehydrogenase-1 (IDH1) mutant protein although *BRAF* testing was not performed at that time. The Ki-67 index was 32%. Post-operatively she received concurrent chemoradiation with a total dose of 60 Gy and temozolomide at 75 mg/m^2^ daily during the course of therapy. Soon after, she received adjuvant therapy as part of a clinical trial with temozolomide at 150 mg/m2 days 1-5, memantine 10 mg twice a day, metformin 500 mg twice a day, and mefloquine 250 mg twice a week (NCT01430351). Due to significant toxicity with severe nausea and fatigue, metformin and, subsequently, mefloquine was discontinued.

Two months after starting adjuvant chemotherapy, restaging scans revealed Progressive Disease (PD) and she was switched to bevacizumab 10 mg/kg every 2 weeks. After one month, capecitabine was added to the regimen at 1250 mg/m^2^ every 2 weeks. She stopped capecitabine due to hand/foot syndrome-related toxicity after 5 months of therapy. She was subsequently started on lomustine at 75 mg/m^2^ on day 3 every 6 weeks, which she received for the next four months. Unfortunately, she eventually developed persistent G2 thrombocytopenia and lomustine was discontinued. She was restarted on bevacizumab monotherapy which she tolerated well for the next 18 months. A re-staging MRI brain at this juncture demonstrated two new dural-based lesions in the right frontal lobe and right occipital lobe. She received radiation therapy to a total dose of 40 Gy in 15 fractions for the recurrent dural lesions in September 2016. Thereafter, she continued maintenance therapy with bevacizumab for one more year.

In September 2017, a restaging MRI Brain demonstrated progression in the afore two dural lesions, one in the right inferior frontal lobe and the other in the right occipital lobe. She underwent a repeat craniotomy with gross total resection and pathology confirmed the diagnosis of recurrent glioblastoma. Next-generation sequencing (NGS) of the tumor showed an actionable *BRAF V600E* mutation and *MET* N375S mutation of germline origin with unknown actionability while immunohistochemistry testing revealed *IDH 1/2* were wild-type.

Based on this *BRAF V600E* mutation, she was genomically matched to enroll on the ROAR basket trial with dabrafenib at 150 mg twice a day plus trametinib at 2 mg daily (NCT02034110). Initially, her best response was stable disease, but after 10 cycles of therapy, the MRI of the brain confirmed a Partial Response (PR). Her best response was a −55% tumor reduction according to the Response Assessment in Neuro-Oncology (RANO) criteria^13^. She tolerated the regimen reasonably well with no dose reductions or treatment interruptions.

**Fig. 2:**
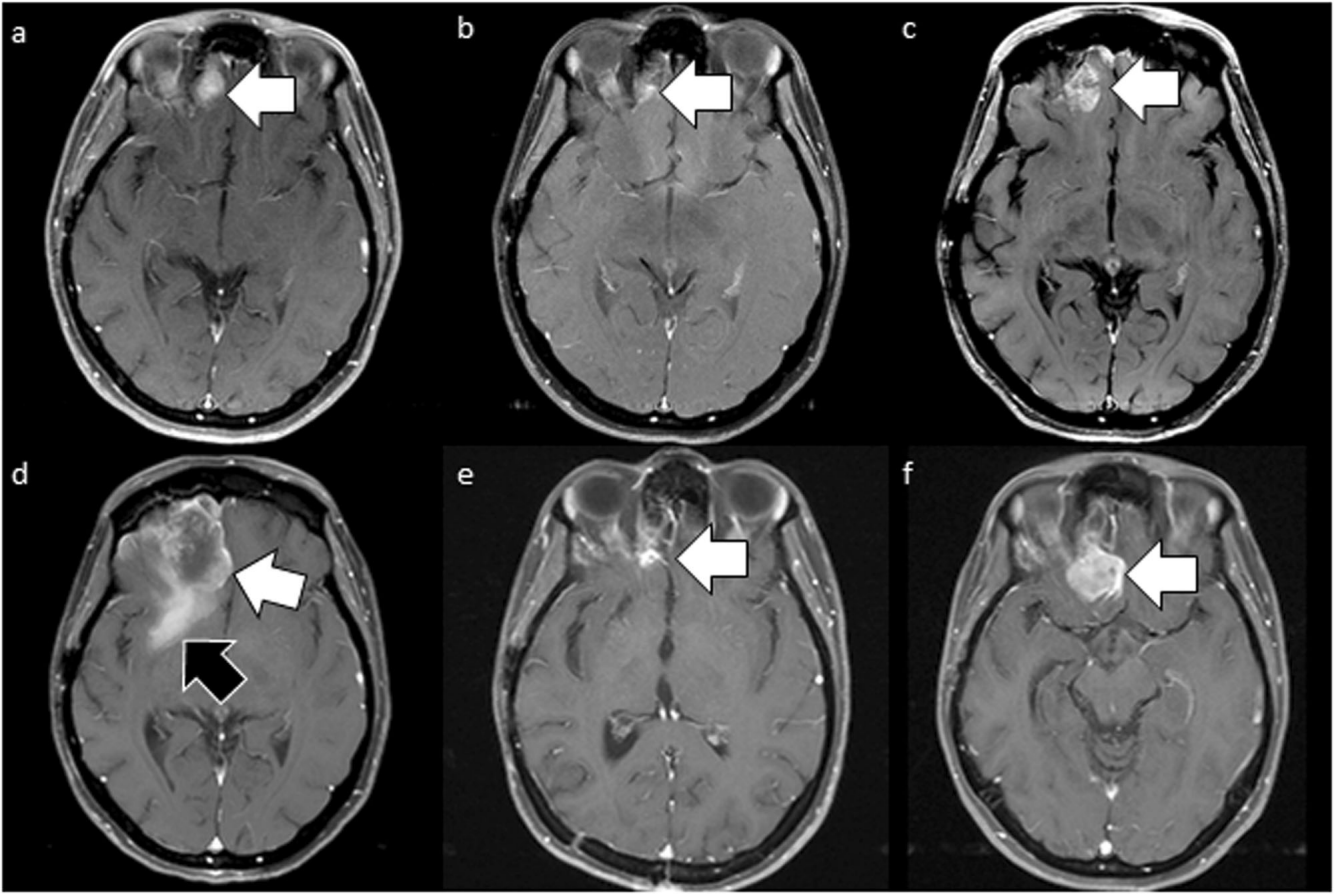
Radiological responses over time. **a** Axial contrast-enhanced MRI while the patient was on single-agent bevacizumab shows a dural-based frontal lobe metastasis (arrow). **b** Axial contrast-enhanced image 9 months after radiation therapy shows significant improvement in the irradiated lesion (arrow). **c** Axial contrast-enhanced MRI 5 months later shows recurrence of the metastasis (arrow). The lesion demonstrates heterogeneous enhancement. **d** Axial post-contrast MRI 2 months later shows the rapid growth of the cystic lesion (white arrow) and extensive perilesional enhancement (black arrow). **e** Axial post-contrast image at treatment nadir 2 months after dabrafenib shows a significant decrease in the size of the lesion. **f** Axial post-contrast image 3 months later shows recurrence of the lesion, this time with solid enhancement (white arrow). The lesion was subsequently resected, showing gliosarcoma.

**Fig. 3:**
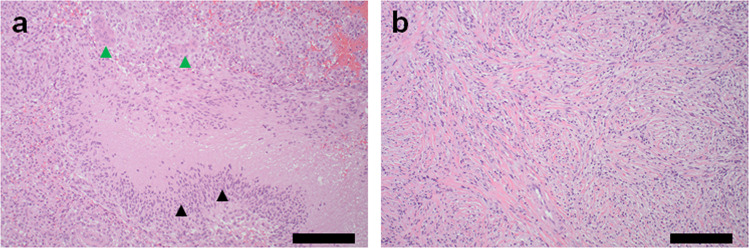
Representative histopathological images. **a** Primary resection showing histopathological features of glioblastoma, including pseudo palisading necrosis (black arrowheads) and microvascular proliferation (green arrowheads). **b** Recurrent resection showing spindle cell sarcomatous histopathology. Scale bars represent 200 μm.

After 18 cycles of dabrafenib and trametinib therapy, a re-staging MRI of the brain revealed a new lesion in the right inferior frontal lobe, while the other lesions remained stable. Her case was discussed in a multidisciplinary tumor board setting, and the patient underwent craniotomy with the gross total resection of the new lesion. The histology of this specimen showed focal transformation to gliosarcoma in the background of glioblastoma. Figure 2 highlights the radiological metamorphosis of patient’s primary glioblastoma to gliosarcoma through the course of therapy. Figure 3 highlights the histopathological differences in the primary glioblastoma and secondary gliosarcoma lesions. Repeat NGS testing on this specimen revealed the emergence of new activating and actionable oncogenic *KRAS* ^*G12D*^ and *NF1* ^*L1083R*^ mutations co-existing with the primary *BRAF V600E* mutation.

She continued therapy with dabrafenib and trametinib for four more months as she was deriving clinical benefit. Subsequent MRI of the brain demonstrated further progression and hence she was taken off trial 3 months later she was referred to the hospice due to clinical deterioration.

## Discussion

*BRAF600* mutations are known to be primary oncogenic drivers in multiple tumors^14^. In glioblastoma, it occurs in a small portion of *IDH*-wild type tumors, corresponding to 8% of the cases^15^. *BRAF*-targeted therapy has set precedence in demonstrating overall and progression-free survival benefits in multiple tumor types harboring the *BRAF V600E* mutation. This led to the agnostic approval of dabrafenib and trametinib by the FDA in June 2022^16,17^. Among adult patients with *BRAF V600E* mutated brain tumors, 15 of the 31 high-grade glioma patients had an objective response rate (ORR) of 33% with 3 Complete Responses (CR) and 12 PRs while the duration of response among all high-grade tumors was 13.6 months. Median progression-free survival was 3·8 months and overall survival was 17·6 months^18^. Notably, this patient benefitted from Dabrafenib and Trametinib for 21.4 months higher than the median survival outcomes as noted before.

In melanoma, resistance mechanisms emerging after treatment with *BRAF*-targeted therapy are well known and they correspond mainly to recovery of *MEK/ERK* signaling or activation of *PI3K/AKT* signaling, through *BRAF* amplification and alternative splicing or alterations in *RAS*, *MEK*, and *ERK*^10^. The mechanism of drug resistance includes alteration of drug targets, expression of drug pumps, expression of detoxification mechanisms, reduced susceptibility to apoptosis through *p53*, increased ability to repair DNA damage, and altered proliferation^19^.

Histologic transformation to small cell lung cancer (SCLC) is a widely known resistance mechanism to epidermal growth factor receptor (EGFR) targeted therapy in Non-Small Cell Lung Cancer (NSCLC) occurring in 3-15% of *EGFR* aberrated NSCLC^20^. Patients who undergo histologic transformation to small cell lung cancers have dismal outcomes. A systemic review looking at outcomes demonstrated a median survival of 6 months after SCLC transformation^21^.

In this patient’s case, acquired mutations *in KRAS* with oncogenic potential and *NF1* with unknown actionability were observed after prolonged exposure to *BRAF* inhibition along with a morphological transformation to gliosarcoma. Gliosarcoma is a rare histopathological variant of *IDH*-wildtype GBM and accounts for ~2% of glioblastoma variants. Histopathologically, these tumors demonstrate a combination of glial areas and sarcomatoid and mesenchymal differentiated components. Secondary gliosarcoma usually evolves after treatment of primary glioblastoma. These tumors are distinct from radiation-induced gliosarcoma which occurs after intracranial radiotherapy in patients without any prior presence of glioblastoma^22^. In our patient, this histological transformation of the right frontal lobe dural lesion to gliosarcoma occurred while on active therapy with dabrafenib and trametinib with initial response and then breakthrough progression with radiological and pathological transformation. It should be noted that on initial diagnosis, the pathology for this tumor raised the possibility of anaplastic pleomorphic xanthoastrocytoma. However, in the 2019 recurrence, this specimen exhibited morphology more consistent with archetypal IDH-wild type glioblastoma. However, the possibility that this tumor may have originated from an anaplastic PXA remains, particularly given the patient’s relatively young age. Furthermore, one must also consider the contiguous situated placement of the lesion’s proximity to the leptomeninges which could be a contributing factor to sarcomatous transformation.

Similar to *BRAF, the RAS* family of genes also works via *Mitogen-activated protein kinases* (*MAPK)* signaling pathways and activates *RAF* and *PI3K* downstream in independent pathways. Receptor tyrosine kinase signaling via Insulin-like growth factor 1 receptor (*IGF1R*) promotes activation of *PI3K* and phosphorylation of *AKT*^23^. This does not affect *MAPK* as is generally thought, however, *MAPK* and *PI3K* pathways jointly regulate *Mcl-1* which is an anti-apoptotic factor that may promote cancer cell survival and growth. Thus, *MAPK* and *IGF-1R* via *PI3K* and *AKT* signaling pathways are both implicated in the development of *BRAF* inhibitor resistance. In one study in *BRAF*-resistant melanoma, it was found that a combination of *MEK* inhibitor with *PI3K* inhibitor led to tumoricidal effects. This study, however, did not observe the development of new mutations after acquired *BRAF* resistance^24^.

Additionally, important to note is that *KRAS* and *BRAF* do not typically co-occur in gliomas, but a common finding in GBM is aberrant *RAS* signaling. One paper looking at factors influencing aberrant *RAS* signaling found that *RAS* and *BRAF* mutations contributed to aberrant *RAS* signaling in a small portion of GBM^25^. In our case, given that initially neither aberrations were present on pathology and sequentially *BRAF* and then *KRAS* and *NF1* were noted, the mechanism through which these mutations were acquired seems to be through reduced susceptibility for apoptosis as well as altered molecular signaling pathways as they are all present in common pathways. One study demonstrated that targeting both pathways through co-inhibition was more efficient in inducing apoptosis than inhibition of each pathway^23^.

To the best of our knowledge and literature review, this is the first case of *BRAF V600E* mutated GBM with the acquisition of *KRAS* ^*G12 D*^ and *NF1* ^*L1083R*^ mutation both in the *RAS/MAPK* pathway and histologic transformation to gliosarcoma as a resistance mechanism to *BRAF/MEK* inhibition. *MAPK* pathway recovery may act as a secondary mechanism of resistance in glioblastomas harboring *BRAF V600E* after the treatment with BRAF inhibitors.

## Methods

### Participant

The patient was treated with dabrafenib and trametinib following enrollment in the phase II study of Efficacy and Safety of the Combination Therapy of Dabrafenib and Trametinib in Subjects With *BRAF V600E* - Mutated Rare Cancers (NCT02034110) after the collection of the written informed consent.

### Materials

Tumor samples were obtained via surgery performed by a neurosurgeon. FFPE specimens derived from fresh tumor biopsies were reviewed by an MD Anderson pathologist to ensure adequate tumor cellularity (≥20%) for analysis. Tumor samples were evaluated using hematoxylin and eosin staining for tumor cellularity. DNA was extracted, purified, and quantified. All procedures were performed in a CLIA-compliant environment. For genomic analysis, the pre-treatment sample was sequenced and subsequently analyzed in the MD Anderson CLIA molecular diagnostic laboratory using the Ion Ampliseq 50-Gene Assay for the detection of mutations in the coding sequence of 50 genes (Thermo Fisher Scientific, MA, USA). DNA was extracted from the recurrent right frontal lobe lesion, in the MD Anderson CLIA molecular diagnostic laboratory utilizing the Oncomine® platform (Thermo Fisher) for the detection of somatic mutations in the coding sequence of 146 cancer-related genes. The radiologic response was assessed according to RANO.

### Reporting summary

Further information on research design is available in the Nature Research Reporting Summary linked to this article.

## Supplementary information

REPORTING SUMMARY

## Data Availability

Samples were sequenced and analyzed in a CLIA-compliant MD Anderson laboratory as described above. The raw sequencing data are not publicly available due to data privacy regulations and restrictions for use of such data, as stated in the study protocol and patient consent form.
