# Supplementary material for: Histological transformation to gliosarcoma with combined BRAF/MEK inhibition in BRAF V600E mutated glioblastoma
Source: NPJ Precis Oncol. 2023 May 25;7:47. doi: 10.1038/s41698-023-00398-5 (PMC10212928; doi:10.1038/s41698-023-00398-5)
Supplement: Supplementary file 1 — REPORTING SUMMARY [file 41698_2023_398_MOESM1_ESM.pdf]

nature portfolio

Corresponding author(s): Dr Vivek Subbiah

Last updated by author(s): Mar 23, 2023

Reporting Summary

Nature Portfolio wishes to improve the reproducibility of the work that we publish. This form provides structure for consistency and transparency in reporting. For further information on Nature Portfolio policies, see our Editorial Policies and the Editorial Policy Checklist.

Statistics

For all statistical analyses, confirm that the following items are present in the figure legend, table legend, main text, or Methods section.

n/a	Confirmed	
☐	☒	The exact sample size (n) for each experimental group/condition, given as a discrete number and unit of measurement
☐	☒	A statement on whether measurements were taken from distinct samples or whether the same sample was measured repeatedly
☒	☐	The statistical test(s) used AND whether they are one- or two-sided<br>*Only common tests should be described solely by name; describe more complex techniques in the Methods section.*
☒	☐	A description of all covariates tested
☒	☐	A description of any assumptions or corrections, such as tests of normality and adjustment for multiple comparisons
☒	☐	A full description of the statistical parameters including central tendency (e.g. means) or other basic estimates (e.g. regression coefficient) AND variation (e.g. standard deviation) or associated estimates of uncertainty (e.g. confidence intervals)
☒	☐	For null hypothesis testing, the test statistic (e.g. F, t, r) with confidence intervals, effect sizes, degrees of freedom and P value noted<br>*Give P values as exact values whenever suitable.*
☒	☐	For Bayesian analysis, information on the choice of priors and Markov chain Monte Carlo settings
☒	☐	For hierarchical and complex designs, identification of the appropriate level for tests and full reporting of outcomes
☒	☐	Estimates of effect sizes (e.g. Cohen's d, Pearson's r), indicating how they were calculated

Our web collection on statistics for biologists contains articles on many of the points above.

Software and code

Policy information about availability of computer code

Data collection	None used
Data analysis	This is a descriptive report of a human participant. The patient was treated with dabrafenib and trametinib following enrollment in the phase II study of Efficacy and Safety of the Combination Therapy of Dabrafenib and Trametinib in Subjects With BRAF V600E - Mutated Rare Cancers (NCT02034110) after the collection of the written informed consent.

For manuscripts utilizing custom algorithms or software that are central to the research but not yet described in published literature, software must be made available to editors and reviewers. We strongly encourage code deposition in a community repository (e.g. GitHub). See the Nature Portfolio guidelines for submitting code & software for further information.

Data

Policy information about availability of data

All manuscripts must include a data availability statement. This statement should provide the following information, where applicable:

- Accession codes, unique identifiers, or web links for publicly available datasets
- A description of any restrictions on data availability
- For clinical datasets or third party data, please ensure that the statement adheres to our policy

Samples were sequenced and analyzed in a CLIA-compliant MD Anderson laboratory as described above. The raw sequencing data are not publicly available due to data privacy regulations and restrictions for use of such data, as stated in the study protocol and patient consent form.

Human research participants

Policy information about studies involving human research participants and Sex and Gender in Research.

Reporting on sex and gender	This is a descriptive report of one female human participant. The patient was treated with dabrafenib and trametinib following enrollment in the phase II study of Efficacy and Safety of the Combination Therapy of Dabrafenib and Trametinib in Subjects With BRAF V600E - Mutated Rare Cancers (NCT02034110) after the collection of the written informed consent.
Population characteristics	Age, sex, number of prior therapies, type of tumor, prior therapies and tumor biology
Recruitment	Patients was recruited at UT MD Anderson Cancer Center through clinic visits were retrospectively reviewed.
Ethics oversight	The research protocol was approved by The University of Texas MD Anderson Cancer Center's Institutional Review Board.

Note that full information on the approval of the study protocol must also be provided in the manuscript.

Field-specific reporting

Please select the one below that is the best fit for your research. If you are not sure, read the appropriate sections before making your selection.

☒ Life sciences ☐ Behavioural & social sciences ☐ Ecological, evolutionary & environmental sciences

For a reference copy of the document with all sections, see nature.com/documents/nr-reporting-summary-flat.pdf

Life sciences study design

All studies must disclose on these points even when the disclosure is negative.

Sample size	The sample size in this descriptive case report was one
Data exclusions	No exclusions
Replication	As a descriptive report, replicationwas not performed
Randomization	As a descriptive report, randomization was not performed
Blinding	As a descriptive report, randomization was not performed

Reporting for specific materials, systems and methods

We require information from authors about some types of materials, experimental systems and methods used in many studies. Here, indicate whether each material, system or method listed is relevant to your study. If you are not sure if a list item applies to your research, read the appropriate section before selecting a response.

Materials & experimental systems

n/a	Involved in the study	
☒	☐	Antibodies
☒	☐	Eukaryotic cell lines
☒	☐	Palaeontology and archaeology
☒	☐	Animals and other organisms
☐	☒	Clinical data
☒	☐	Dual use research of concern

Methods

n/a	Involved in the study	
☒	☐	ChIP-seq
☒	☐	Flow cytometry
☒	☐	MRI-based neuroimaging

Clinical data

Policy information about clinical studies

All manuscripts should comply with the ICMJE guidelines for publication of clinical research and a completed CONSORT checklist must be included with all submissions.

Clinical trial registration	https://clinicaltrials.gov/ct2/show/NCT02034110
Study protocol	NCT02034110
Data collection	Data was collected at UT MD Anderson Cancer Center
Outcomes	Clinical outcomes with clinical response)that were not predefined were assessed using a descriptive fashion. Due to the nature of this observation study, there are no predefined primary and secondary endpoints.
